# Intergenerational Influence of Gender and the DM1 Phenotype of the Transmitting Parent in Korean Myotonic Dystrophy Type 1

**DOI:** 10.3390/genes13081465

**Published:** 2022-08-17

**Authors:** Ji Yoon Han, Woori Jang, Joonhong Park

**Affiliations:** 1 Department of Pediatrics, College of Medicine, The Catholic University of Korea, Seoul 06591, Korea; 2 Department of Laboratory Medicine, Inha University School of Medicine, Incheon 22332, Korea; 3 Department of Laboratory Medicine, Jeonbuk National University Medical School and Hospital, Jeonju 54907, Korea; 4 Research Institute of Clinical Medicine of Jeonbuk National University, Biomedical Research Institute of Jeonbuk National University Hospital, Jeonju 54907, Korea

**Keywords:** intergenerational influence, gender, DM1 phenotype, CTG repeat, *DMPK* gene, myotonic dystrophy type 1

## Abstract

Myotonic dystrophy type 1 (DM1) is the most common autosomal-dominant disorder caused by the CTG repeat expansion of the *DMPK*, and it has been categorized into three phenotypes: mild, classic, and congenital DM1. Here, we reviewed the intergenerational influence of gender and phenotype of the transmitting parent on the occurrence of Korean DM1. A total of 44 parent–child pairs matched for the gender of the transmitting parent and the affected child and 29 parent–child pairs matched for the gender and DM1 phenotype of the transmitting parent were reviewed. The CTG repeat size of the *DMPK* in the affected child was found to be significantly greater when transmitted by a female parent to a female child (DM1-FF) (median, 1309 repeats; range, 400–2083) than when transmitted by a male parent to a male child (650; 160–1030; *p* = 0.038 and 0.048 using the Tukey HSD and the Bonferroni test) or by a male parent to a female child (480; 94–1140; *p* = 0.003). The difference in the CTG repeat size of the *DMPK* between the transmitting parent and the affected child was also lower when transmitted from a male parent with classic DM1 (−235; −280 to 0) compared to when it was transmitted from a female parent with mild DM1 (866; 612–905; *p* = 0.015 and 0.019) or from a female parent with classic DM1 (DM1-FC) (605; 10–1393; *p* = 0.005). This study highlights that gender and the DM1 phenotype of the transmitting parent had an impact on the CTG repeat size of the *DMPK* in the affected child, with greater increases being inherited from the DM1-FF or DM1-FC situations in Korean DM1.

## 1. Introduction

Myotonic dystrophy type 1 (DM1; OMIM #160900) is the most common type of muscular dystrophy in adults with a total age-standardized prevalence of 9.65/100,000 in the Rome province. It is mainly characterized by a multi-systemic progressive disease with symptoms such as muscular dystrophy, myotonia, hypogonadism, cataracts, and gastrointestinal, central nervous system, endocrine, cardiac conduction, and skin defects. DM1 is transmitted in an autosomal-dominant manner and results from the pathologic expansion of a polymorphic CTG repeat in the 3′ untranslated region of the *DMPK*, which encodes for the dystrophia myotonica protein kinase (OMIM * 605377). Patients with DM1 harbor alleles with expanded CTG repeats, ranging from 50 to >1000 CTG repeats, and have been categorized into three phenotypes correlating with their CTG repeat expansion size in the *DMPK*: mild, classic, and congenital DM1 [1,2,3]. The CTG repeat expansion of the *DMPK* shows both intergenerational and mitotic instability that are biased toward expansion. This results in interindividual variability due to the phenomena of anticipation and tissue mosaicism during linear transmission [4]. Several studies have reported that the CTG repeat length of the *DMPK* in peripheral leukocytes correlates inversely with the age of disease onset and is statistically correlated with both grip strength and myotonia [5,6].

The CTG repeats in the *DMPK* may further increase in length during gametogenesis, leading to the transmission of an allele with an expanded CTG repeat region that may be associated with a more severe DM1 phenotype and earlier onset of disease in the affected child [7,8]. There are two paternal factors that influence the degree of mutation expansion in the affected child: the gender of the transmitting parent and the repeat size in the transmitting parent. The mean intergenerational expansion is statistically high when DM1 is transmitted by a female; however, it is minimal when transmitted by a male regardless of the parent’s expansion size [8]. Other reports have found that patients born to affected mothers have the same tendency to show a CTG repeat expansion of the *DMPK* as those born to affected fathers [9,10]. On the other hand, a correlation between the frequency of congenital DM1 and the maternal CTG repeat size has been suggested [11]. In contrast, in the affected children of transmitting parents with small expansions (up to 100 CTG repeats), those with expanded alleles inherited paternally were found to have a larger CTG repeat expansion than those with maternally inherited expanded alleles [12].

Several studies have attempted to explain this discrimination and the clinical and genetic variable results, whereby the gender of the transmitting parent influences the CTG repeat size of the *DMPK* in the affected child, causing preferential transmission [13,14,15,16]. However, the gender and the DM1 phenotype of the transmitting parent have not been specifically studied in relation to Korean DM1. Here, we reviewed the intergenerational influence of the gender and phenotype of the transmitting parent with Korean DM1.

## 2. Intergenerational Data Collection and Analysis

Data on the gender, the CTG repeat size of the *DMPK*, and/or the DM1 phenotype of the transmitting parent and the affected child were collected to estimate the intergenerational influence of the gender and DM1 phenotype of the transmitting parent on the development of Korean DM1. The DM1 phenotype was classified as mild DM, classic DM1, or congenital DM. Mild DM1 was defined as cases with a CTG repeat size of 50–100 repeats displaying cataract or mild myotonia such as sustained muscle contraction. Classic DM1 was defined as cases with a CTG repeat size of 100–1000 repeats having cataract, cardiac conduction abnormalities, myotonia, muscle weakness, or wasting. Congenital DM1 was defined as cases with a CTG repeat size of >1000 repeats showing severe generalized weakness or hypotonia at birth, respiratory insufficiency, or intellectual disability. These data were clarified after combining clinical manifestations and molecular analysis results from our patients with the previous studies described. Data from eight patients from five unrelated families with DM1—confirmed by southern analysis after long-range PCR—were initially estimated at the Department of Pediatrics, Daejeon St. Mary’s Hospital (Daejeon, Korea). Southern analysis after long-range PCR was applied to patients referred for the genetic testing of DM1 at the Medical Genetics Center in the Asan Medical Center Children’s Hospital (Seoul, Korea). After pedigree analysis, five parent–child matched pairs with information about each gender and/or repeat size of *DMPK* were enrolled in this study.

In order to collect intergenerational data reported from previous studies, electronic journal databases, including PubMed (https://www.ncbi.nlm.nih.gov/pubmed, accessed on 2 June 2022) and KoreaMed (http://koreamed.org, accessed on 2 June 2022), containing information on the molecular genetics of DM1, were searched from 1990 to 2017. The following terms were used in search strategies: myotonic, dystrophy, type 1, and Korean. All related citations were retrieved to find other relevant articles that were not identified in the initial research. The literature search included Korean as well as English articles. Only reported cases with well-documented relevant information about gender, CTG repeat size of the *DMPK*, and/or the DM1 phenotype of the transmitting parent and the affected child were included. After a literature search related to Korean DM1 [17,18,19,20,21,22,23,24,25,26], 39 parent–child matched pairs with information about each gender, the CTG repeat size of the *DMPK*, and/or the DM1 phenotype were included in this study.

A one-way analysis of variance (ANOVA) with Tukey’s HSD and the Bonferroni test was used to evaluate the child’s CTG repeat size of the *DMPK* and the difference in the CTG repeat size of the *DMPK* between the transmitting parent and the affected child in four groups categorized according to the gender of the transmitting parent and the affected child and the gender and DM1 phenotype of the transmitting parent. The statistical analyses were carried out using MedCalc ver. 12.7.2 (MedCalc software, Mariakerke, Belgium), and a *p* value of <0.05 was considered to be statistically significant.

## 3. Intergenerational Influences in Korean DM1

A total of 44 parent–child pairs matched according to the gender of the transmitting parent and the affected child and 29 parent–child pairs matched according to the gender and the DM1 phenotype of the transmitting parent were used to estimate the intergenerational influence of the gender and DM1 phenotype of the transmitting parent on the development of Korean DM1.

To estimate the intergenerational influence of the gender of the transmitting parent and the affected child, 44 parent–child matched pairs were compared using four groups based on the gender of the transmitting parent and the affected child. As a result, maternal transmission (*n* = 28) was found to be 1.75 times more common than paternal transmission (*n* = 16) (Table 1).

**Figure 1:**
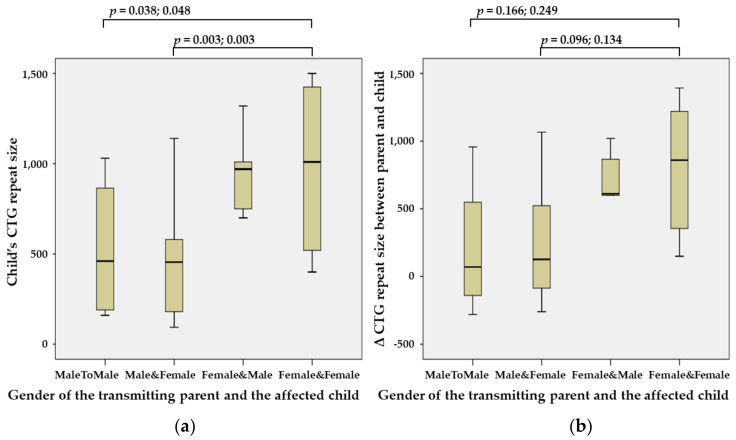
Intergenerational influence of gender between the transmitting parent and the affected child. (**a**) The CTG repeat size of the *DMPK* in the affected child was significantly greater when the *DMPK* repeats were transmitted from a female parent to a female child (DM1-FF) (median, 1309 repeats; range, 400–2083) than when they were transmitted from a male parent to a male child (DM1-MM) (650; 160–1030; *p* = 0.038 and 0.048 using the Tukey HSD and the Bonferroni test) and from a male parent to a female child (DM1-MF) (480; 94–1140; *p* = 0.003). (**b**) The difference in the CTG repeat size of the *DMPK* between the transmitting parent and the affected child was also higher in DM1-FF than in DM1-MM and in DM1-MF, but this result was not statistically significant.

The CTG repeat size of the *DMPK* in the affected child was significantly greater when the *DMPK* repeats were transmitted from a female parent to a female child (DM1-FF) (median, 1309 repeats; range, 400–2083) than when they were transmitted from a male parent to a male child (DM1-MM) (650; 160–1030; *p* = 0.038 and 0.048 using the Tukey HSD and the Bonferroni test) or from a male parent to a female child (DM1-MF) (480; 94–1140; *p* = 0.003) (Figure 1a). The difference in the CTG repeat size of the *DMPK* between the transmitting parent and the affected child was also higher in DM1-FF than in DM1-MM or DM1-MF, but this finding was not statistically significant (Figure 1b).

**Figure 2:**
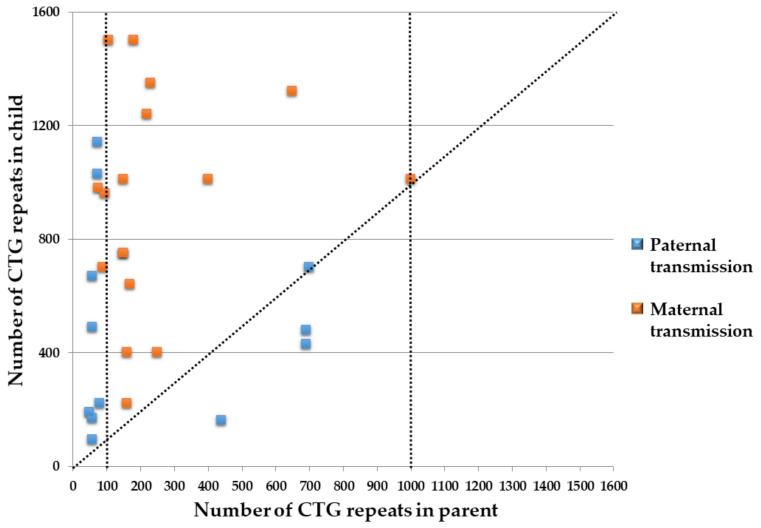
The influence of the CTG repeat size of the *DMPK* of the transmitting parent on the affected child. CTG repeat reduction or no expansion only occurred following paternal transmission, even though the transmitting father and the affected child were diagnosed as having classic DM1. The vertical dotted line is used to indicate the mild (CTG repeat size with 50–100 repeats), classic (100–1000), and congenital (>1000) DM1 phenotypes. The diagonal dotted line indicates the influence of the CTG repeat size of the *DMPK* of the transmitting parent on that of the affected child.

To evaluate the intergenerational influence of the gender and DM1 phenotype of the transmitting parent, 29 parent–child matched pairs were compared using four groups based on the gender and DM1 phenotype of the transmitting parent. The results show that the CTG repeat expansion of the *DMPK* always occurred when transmitted from a parent with mild DM1 regardless of their gender. On the contrary, the CTG repeat reduction or non-expansion of the *DMPK* only occurred when the condition was transmitted paternally, even though the transmitting father and the affected child were diagnosed with classic DM1 (Figure 2). However, two-thirds (62%, 18/29) of the affected children were found to have a more severe DM1 phenotype than their parent (Table 2).

**Figure 3:**
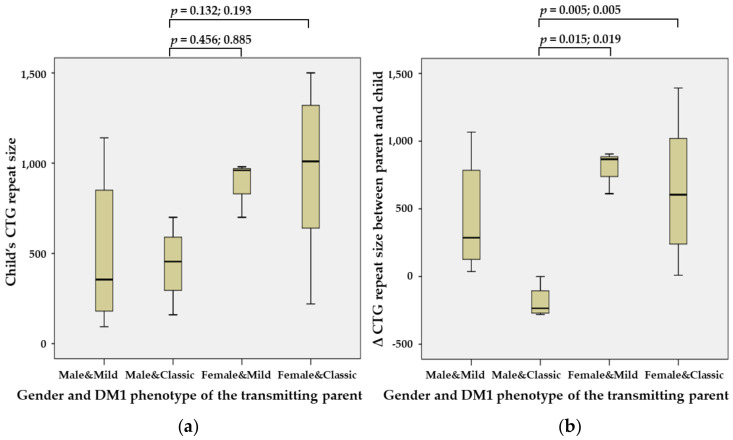
Intergenerational influence of the gender and DM1 phenotype of the transmitting parent. (**a**) The influence of the CTG repeat size of the *DMPK* of the affected parent on the child was lower when the transmitting parent was male and when the condition was classic DM1 (DM1-MC) (455; 160−700) than for maternal transmission and mild DM1 (DM1-Fm) (960; 700–980) or for maternal transmission and classic DM1 (DM1-FC) (1010; 220–1500). (**b**) The difference in the CTG repeat size of the *DMPK* between the transmitting parent and the affected child was also lower in DM1-MC (−235; −280 to 0), compared with DM1-Fm (866; 612–905; *p* = 0.015 and 0.019) and DM1-FC (605; 10–1393; *p* = 0.005).

The difference in the CTG repeat size of the *DMPK* between the transmitting parent and the affected child was lower when transmitted from a male parent with classic DM1 (DM1-MC) (455; 160–700) than when transmitted from a female parent with mild DM1 (DM1-Fm) (960; 700–980) or a female parent with classic DM1 (DM1-FC) (1010; 220–1500), although this result was not statistically significant. The difference in the CTG repeat size of the *DMPK* between the transmitting parent and the affected child was also lower in DM1-MC (−235; −280–0) compared with DM1-Fm (866; 612–905; *p* = 0.015 and 0.019) and DM1-FC (605; 10–1393; *p* = 0.005) (Figure 3).

## 4. Discussion

Geographically, large-scale population-based studies have been reported in Asia [27,28,29,30], Europe [31,32,33,34,35,36,37,38,39,40], North America [41], and Oceania [42]. Previous studies have reported that the incidence of DM1 is different in various ethnicities [43,44,45]. DM1 is more frequent in a European population; however, it is very rare in a Southern African population [44,45]. In an Asian population, the estimated incidence of DM1 was found to be low in Taiwan [28] with 0.45 patients per 100,000. The low incidence of DM1 in a Taiwanese population may be explained by the number of the CTG repeats of the *DMPK* with <18 [46]. In addition, the clinical manifestations of Chinese DM1 patients are distinguished from those of Caucasian DM1 patients [47], emphasizing the importance of appropriate molecular analysis for the diagnosis of DM1. Recently, Nicholas et al. demonstrated that founder effects are not a likely cause for the increased occurrence in which individuals with the CTG repeat expansion of the *DMPK* with ≥50 were not closely related and have diverse genetic ancestry, even though the incidence of DM1 may be higher in some populations than others due to founder effects [48]. Furthermore, the incidence of individuals with CTG repeat expansions of the *DMPK* is up to 5 times higher than previously reported estimates. These findings suggest that DM1, with multi-systemic characteristics, is likely underdiagnosed in practice [48].

We reviewed the intergenerational influence of the gender and DM1 phenotype of the transmitting parent on the development of Korean DM1. First, maternal transmission was related to the CTG repeat size of the *DMPK* and to the more severe DM1 phenotype, as shown in previous studies [2,8,14]. In children with congenital DM1, the condition is more frequently inherited maternally (*n* = 16) rather than paternally (*n* = 2). This phenomenon has been attributed to substantial DNA instability in the female germ cell lineage, leading to additional CTG repeat insertion during oogenesis [49]. DNA instability results in anticipation during maternal transmission, an occurrence corresponding to more severe DM1 phenotypes and earlier disease onset in consecutive generations. In contrast to our results, Dogan et al. reported that maternal transmission was observed in a minority of DM1 patients (37%). This probably resulted from perinatal lethality and increased miscarriage found in maternal DM1 transmitters [14].

Second, the gender difference suggested an unequal prevalence of several DM1 phenotypes in the affected child. Interestingly, paternal transmission (*n* = 8) was found to be 2.67 times more common than maternal transmission (*n* = 3) when the transmitting parent showed the mild DM1 phenotype in this study. However, this difference could not be explained by differences in the CTG repeat size of the *DMPK* or by male-to-female disproportion between the transmitting parent and the affected child in this study. The exact process underlying gender-dependent differences is unknown. Different characteristics of skeletal muscle tissue between males and females have been associated with significant differences in gene expression patterns and metabolic properties [50]. In myotonia congenita, sexual steroid hormones reflect testosterone modulation of CLCN1 chloride channel activity and affect clinical manifestations of myotonia congenita [51]. CTG repeat reduction and expansion of the *DMPK* could occur during the intergenerational transmission of *DMPK* expansion with variant CTG repeats in DM1 families. Cases of *DMPK* expansion with 50–100 CTG repeats are generally stable in maternal transmission and expansion frequently occurs in paternal transmission [52]. Compared with the maternal transmission of small-sized CTG repeats, the paternal transmission of DM1 pre-(CTG repeat size with 36–50 repeats) and proto-(CTG repeat size with 51–80 repeats) mutations is far more unstable [53]. This emphasizes that males with a small *DMPK* mutation have a higher risk of symptomatic offspring compared with females.

Third, *DMPK* expansions with >100–200 CTG repeats are volatile and are transmitted by both genders. In this size range, transmissions largely lead to CTG expansions of the *DMPK*; however, cases of stable CTG repeat reduction and expansion can occur, especially in paternal transmissions [54,55]. Large *DMPK* expansion with >1000 CTG repeats was found in immature and metaphase II oocytes; however, these large, mutated alleles were absent in the sperm of most male DM1 patients, consistent with the low frequency of paternal transmissions of congenital DM1 [49,56]. Methylation of the sequence located around the expanded CTG repeat of the *DMPK* might explain the maternal bias for the transmission of large expansions and congenital DM1, to a certain degree. A recent hypothesis to explain the almost exclusive maternal transmission of congenital DM1 suggests the prevention of the transmission of large repeat expansions after paternal transmissions, leading to the reduced expression of the *SIX5* gene in the DM1 locus [57]. However, Yanovsky-Dagan et al. exclude the possibility that *DMPK* hypermethylation leads to selection against viable sperm cells in DM1 patients by assessing DNA methylation upstream to the CTG expansion of the *DMPK* in motile sperm cells of four DM1 patients [58]. Nevertheless, CTG repeat expansion of the *DMPK* harboring a single CAG interruption is characterized by stable transmission or CTG repeat reduction in successive generations [59], suggesting that a single nucleotide change within the CTG repeat may be enough to strengthen the meiotic stability of *DMPK* expansion. Although the transmission of interrupted *DMPK* expansions was not associated with the gender of the transmitting parent [60], it is noteworthy that all sporadically occurring variant *DMPK* expansions were transmitted by the male parent, whether it was an expansion harboring different patterns of CCG repeats [61,62] or a single CTC repeat [60].

A limitation of this study is the small study cohort of 44 patients and reported cases; nevertheless, the uniform clinical characterization of the participants, including their gender, the CTG repeat size of the *DMPK*, and/or the DM1 phenotype, is a strength of this study. Another limitation is the lack of detailed clinical information. The pathogenesis of DM1 is complex, with a pivotal role played by the pathogenic effect of the mutant *DMPK* pre-mRNAs harboring the expanded CUG stretch, which will eventually interrupt the expression of other genes in different tissues by damaging the functions of specific transcription factors controlling alternative splicing. Because the *DMPK* mRNA is expressed widely in various tissues, this finding explains the multi-organ involvement in patients with DM1, including the associated development of gastrointestinal disturbances, endocrine dysfunction, cardiac conduction abnormalities, premature cataracts, and behavioral and cognitive impairments [15]. Male patients with DM1 tended to show more apparent classical DM1 phenotypes, such as cardiac and respiratory involvement, significant myotonia, and cognitive impairment; however, female patients with DM1 had late-onset disease and more extra-muscular clinical features, characteristics which are less suggestive of DM1. These findings emphasize the significance of having greater recognition of preventive medical management in male individuals with DM1 [14]. Thus, the gender-associated differential risks of developing certain symptoms may require gender-orientated therapeutic care. In addition, we could not estimate the presence of common lifestyle risk factors in our cohort that may affect the risk of cancer in DM1, as reported previously [63].

## 5. Conclusions

In conclusion, the gender and DM1 phenotype of the transmitting parent impact the CTG repeat size of the *DMPK* in the affected child, with higher increases being inherited from DM1-FF or DM1-FC situations in Korean DM1. Thus, the gender and DM1 phenotype of the transmitting parent should be considered in the design of both stratified clinical trials and medical management. Further investigations are required to clarify the complex pathophysiology associated with the gender and DM1 phenotype of the transmitting parent and the affected child in order to make specific recommendations regarding the diagnostic assessment and medical care of the different DM1 phenotype distributions of Korean DM1 patients, as this often affects the quality of life of patients with DM1.

## Figures and Tables

**Table 1:** DM1 phenotypes and CTG repeat size of the *DMPK* in the affected child according to the gender of the transmitting parent and the affected child in 44 Korean parent–child matched pairs diagnosed with DM1.

Parent Gender	Child Gender	Child DM1 Phenotype			Child’s Repeat Size; Median (Range)	Δ Repeat Size; Median (Range)
		Mild	Classic	Congenital		
Male	Male	0	4	1	650 (160–1030)	70 (−280–957)
Male	Female	1	9	1	480 (94–1140)	127 (−260–1067)
Female	Male	0	8	6	970 (220–1667)	611 (10–1020)
Female	Female	0	4	10	1309 (400–2083)	860 (150–1393)

Δ Repeat size, difference between the transmitting parent and the affected child.

**Table 2:** DM1 phenotypes and CTG repeat size of the *DMPK* in the affected child according to gender and DM1 phenotype of the transmitting parent in 29 Korean parent–child matched pairs diagnosed with DM1.

Parent Gender	Parent Phenotype	Child DM1 Phenotype			Child’s Repeat Size; Median (Range)	Δ Repeat Size; Median (Range)
		Mild	Classic	Congenital		
Male	Mild	1	5	2	355 (94–1140)	287 (37–1067)
Male	Classic	0	4	0	455 (160–700)	−235 (−280–0)
Female	Mild	0	3	0	960 (700–980)	866 (612–905)
Female	Classic	0	6	8	1010 (220–1500)	605 (10–1393)

Δ Repeat size, difference between the transmitting parent and the affected child.

## Data Availability

Not applicable.
